# Heat Shock Protein 90 Regulates the Activity of Histone Deacetylase Sir2 in Plasmodium falciparum

**DOI:** 10.1128/msphere.00329-22

**Published:** 2022-09-19

**Authors:** Wahida Tabassum, Mrinnanda Bhattacharya, Sayan Bakshi, Mrinal Kanti Bhattacharyya

**Affiliations:** a Department of Biochemistry, School of Life Sciences, University of Hyderabad, Hyderabad, India; b Department of Systems and Computational Biology, School of Life Sciences, University of Hyderabad, Hyderabad, India; Indiana University School of Medicine

**Keywords:** PfSir2, PfHsp90, chaperone, *var* gene expression, antigenic variation, malaria, rDNA silencing

## Abstract

Sir2 protein of Plasmodium falciparum has been implicated to play crucial roles in the silencing of subtelomeric *var* genes and rRNA. It is also involved in telomere length maintenance. Epigenetic regulation of *PfSIR2* transcription occurs through a direct participation of the molecular chaperon PfHsp90, wherein PfHsp90 acts as a transcriptional repressor. However, whether the chaperonic activity of PfHsp90 is essential for the maturation and stability of PfSir2A protein has not yet been explored. Here, we show that PfSir2A protein is a direct client of PfHsp90. We demonstrate that PfHsp90 physically interacts with PfSir2A, and the inhibition of PfHsp90 activity via chemical inhibitors, such as 17-AAG or Radicicol, results in the depletion of PfSir2A protein, and consequently its histone deacetylase activity. Thus, derepression of *var* genes and ribosomal silencing were observed under PfHsp90 inactivation. This finding that PfHsp90 provides stability to PfSir2A protein, in addition to the previous finding that PfHsp90 downregulates *PfSIR2A* transcription and subsequently cellular abundance, uncovers the multifaceted roles of PfHsp90 in regulating PfSir2 abundance and activity. Given the importance of PfSir2 protein in *Plasmodium* biology, it is reasonable to propose that the PfHsp90-PfSir2 axis can be exploited as a novel druggable target.

**IMPORTANCE** Malaria continues to severely impact the global public health not only due to the mortality and morbidity associated with it, but also because of the huge burden on the world economy it imparts. Despite the intensive vaccine-research and drug-development programs, there is not a single effective vaccine suitable for all age groups, and there is no drug on the market against which resistance is not developed. Thus, there is an urgent need to develop novel intervention strategies by identifying the crucial targets from *Plasmodium* biology. Here, we uncover that the molecular chaperone PfHsp90 regulates the abundance and activity of the histone-deacetylase PfSir2, a prominent regulator of *Plasmodium* epigenome. Given that PfSir2 controls both virulence and multiplicity of the parasite, and that PfHsp90 is an essential chaperone involved in diverse cellular processes, our findings argue that the PfHsp90-PfSir2 axis could be targeted to curb malaria.

## INTRODUCTION

The molecular chaperone heat shock protein 90 (Hsp90) plays a pivotal role in the maturation of a subset of the eukaryotic proteome, known as its clients. Unlike Hsp70 and Hsp40 that are involved in the general folding of the nascent polypeptide chains, Hsp90 functions at a later stage and its action is limited to its clientele. In the model organism Saccharomyces cerevisiae, interaction of Hsp90 is restricted to only 10% of the proteome (1, 2). The interactome of Hsp90 includes several signaling kinases, DNA repair proteins, steroid-hormone receptors and other transcription factors, chromatin remodellers, and telomere maintenance proteins, including telomerase (3–6).

There are two paralogues of *HSP90* gene in yeast and humans. In these organisms, the simultaneous knockout of both the paralogues is lethal, suggesting that Hsp90 is essential for survival (7, 8). Hsp90 exists as a homodimer via interaction through its C-terminal domain. The N-terminal ATPase domain is crucial for its canonical chaperoning function of client maturation (9, 10). This process also requires the participation of several cochaperones of Hsp90 (3).

The human malaria parasite Plasmodium falciparum possesses four paralogues of Hsp90. Out of the two cytosolic PfHsp90 proteins, one is constitutive (PF3D7_1443900) and the other is heat inducible (PF3D7_0708400). The other two paralogues (PF3D7_1118200, and PF3D7_1222300) are present in the mitochondria and ER, respectively. A forward genetics screen with *piggyback* transposons predicted the essential nature of *PfHSP90* genes, as all the four paralogues of *PfHSP90* genes were found to be devoid of transposon insertions in their CDS (11). *In vitro* studies with purified recombinant PfHsp90 protein revealed that the inducible form of the protein (PF3D7_0708400) could be inhibited by a small-molecule inhibitor 17-AAG (12). Additionally, functional inhibition of PfHsp90 by another chemical, Radicicol has also been observed in yeast surrogate system (13). Both the chemicals have also exhibited anti-plasmodial activity in *in vitro* culture system (14–16). The list of PfHsp90 clientele remained enigmatic. Recently, the *Plasmodium* recombinase PfRad51 has been established as a client of PfHsp90 (17). Inhibition of PfHsp90 abrogated DNA-double-strand break repair in this parasite and rendered *Plasmodium* hypersensitive to DNA-damaging agents (17). An extreme synergism was also observed between the PfHsp90 and PfRad51 inhibitors (17).

Sir2 is a NAD+-dependent histone deacetylase and is involved in regional gene silencing mechanism. This has been implicated in mating-type silencing, telomere position effect (TPE), and rDNA silencing in budding yeast (18). It is also known to prevent aberrant recombination between rDNA repeats (19). In higher organisms, Sirtuins, the orthologues of yeast Sir2, also play important roles in DNA repair involving NHEJ pathway (20, 21). Moreover, in higher organisms, several nonhistone clients of Sirtuins have also been identified (22).

In P. falciparum, there are two paralogues of Sirtuins: PfSir2A and PfSir2B. Both of these have been implicated in the silencing of the subtelomeric *var* genes, where each of them regulates different sets of *var* genes (23). Thus, PfSir2 ensures mono-allelic expression of *var* genes, which is the most crucial step during the maintenance of antigenic variation in this parasite. Therefore, the subtelomeric gene silencing function of *Plasmodium* Sir2 protein contributes toward the virulence of the parasite. PfSir2 has also been implicated in the transcriptional silencing of the nucleolar r-RNA genes. The role of PfSir2 in rRNA synthesis is linked to the numbers of daughter merozoites, and hence this particular function of PfSir2 influences the multiplication rate of the parasite (24). Although no roles of PfSir2 in *Plasmodium* DNA repair have been reported, its role in the maintenance of telomere length is well established (23). From literature we know that PfSir2A predominantly deacetylates Histone H3 at K9 position, and possibly H4Ac as well (23, 25); however, any non-Histone clients of PfSir2 have not yet been identified.

Apart from its cytoplasmic canonical function of client maturation, Hsp90 also exhibits noncanonical nuclear functions in model organisms. Such moon-lighting functions include nuclear import of proteins, assembly and disassembly of proteins on chromatin, and several aspects of chromatin remodeling (6). In the budding yeast it has been found that Hsp90 modulates the transcriptional fate of *SIR2* by nuclear proteostasis, where it controls the level of the transcription factor Cup9 (26). In P. falciparum, PfHsp90 acts as a transcriptional repressor of *PfSIR2A/B* gene expression. Under overexpression conditions, PfHsp90 occupies the promoters of *PfSIR2A/B* and remodels the associated chromatin into the compact heterochromatin state, leading to the downregulation of *PfSIR2* transcription (27). Such processes were found to be tightly regulated in a developmental stage specific manner. Low abundance of PfSir2 transcript and protein led to a reduction in the PfSir2 function at the subtelomeric *var* loci, resulting in the derepression of *var* transcription (27).

However, whether an inhibition of PfHsp90 function has any effect on the cellular abundance and function of PfSir2 remains unexplored. Here, we show that inhibition of PfHsp90 function, with two structurally unrelated chemical inhibitors, leads to the depletion of PfSir2A protein, consequently its deacetylase activity on Histone H3 at K9 position, and PfSir2 functions at both the nucleolar rDNA locus and the subtelomeric *var* loci. We further demonstrate direct physical interaction between PfHsp90 and PfSir2, establishing the clientship, as proposed earlier.

## RESULTS

### Inhibition of PfHsp90 results in the derepression of the *var* genes and the rRNA genes.

**FIG 1:**
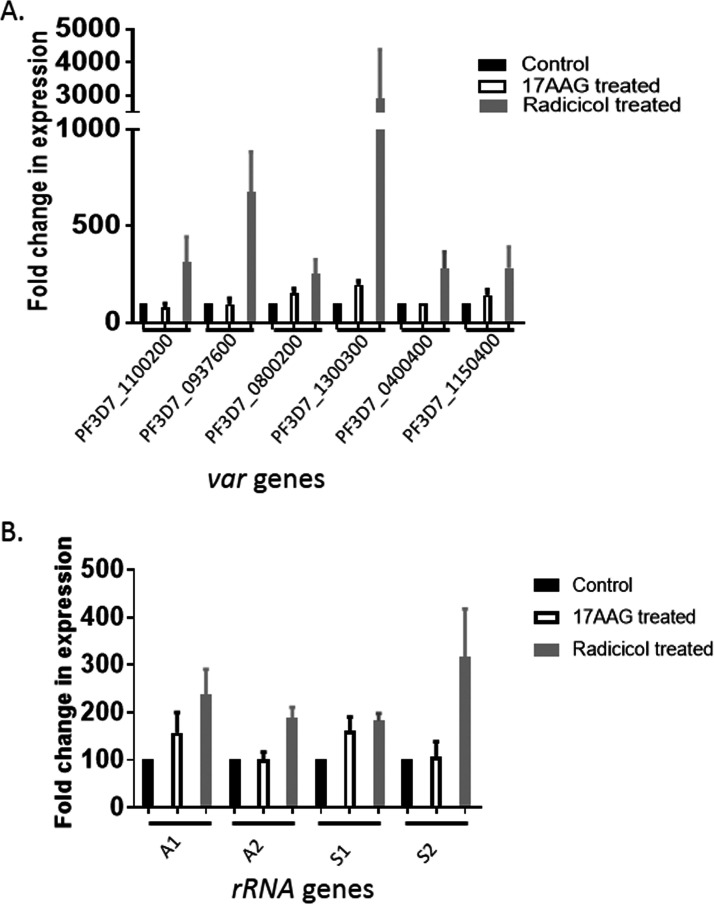
Inhibition of PfHsp90 results in the derepression of the *var* genes and the rRNA genes. (A) The graph depicts the transcription of *var* genes belonging to UpsA at normal conditions and the change in its transcription upon Hsp90 inhibition by 1.7 μM 17-AAG or 8.5 μM Radicicol, quantified through real-time RT-PCR. The Gene ID of each *var* gene is marked at the bottom. (B) The graph shows the transcription of rRNA genes (A1, A2, S1, S2) at normal conditions and alteration in its expression upon Hsp90 inhibition by 17-AAG and Radicicol, quantified through real-time RT-PCR. The expression level of *seryl-tRNA synthetase* was used for normalization for both the graphs. Fold change with respect to the corresponding untreated control is plotted for each gene. The qPCR results are representative of three independent experiments with data indicating the mean ±SEM.

As the histone de-acetylase activity of PfSir2 has been implicated in the transcriptional repression of the subtelomeric *var* genes, and in the nucleolar rRNA genes, we investigated the transcriptional status of these genes in order to investigate whether inhibition of PfHsp90 activity leads to the functional inactivation of PfSir2. Six *UPS-A* type *var* genes that are exclusively controlled by PfSir2A protein were included in our study. To inactivate PfHsp90 activity, we used two structurally dissimilar well-known chemical inhibitors, namely, 17-AAG and Radicicol. All the six *var* genes tested were derepressed by 1.5-fold or more upon 8.5 μM Radicicol treatment. A similar but less pronounced effect was observed in the case of 1.7 μM 17-AAG treatment (Fig. 1A). The maximum derepression was observed for *var* gene PF3D7_1300300. The plausible reason for such a high-level of derepression of this particular gene is currently unknown. Similarly, upon Radicicol treatment, the expressions of all the four rRNA genes were found to be upregulated by 1.5-fold or more. The extent of derepression upon 17-AAG treatment was found to be less prominent (Fig. 1B). It could be possible that the dose of 17-AAG treatment may not be optimum to achieve as good inhibition of PfHsp90 as obtained by Radicicol treatment. Nonetheless, the derepression of the majority of the genes known to be silenced by PfSir2A, in response to two independent inhibitors of PfHsp90, suggests a strong connection between PfHsp90 activity and PfSir2 function.

### Chemical inhibition of PfHsp90 leads to hyperacetylation of histone H3 at K9 position.

**FIG 2:**
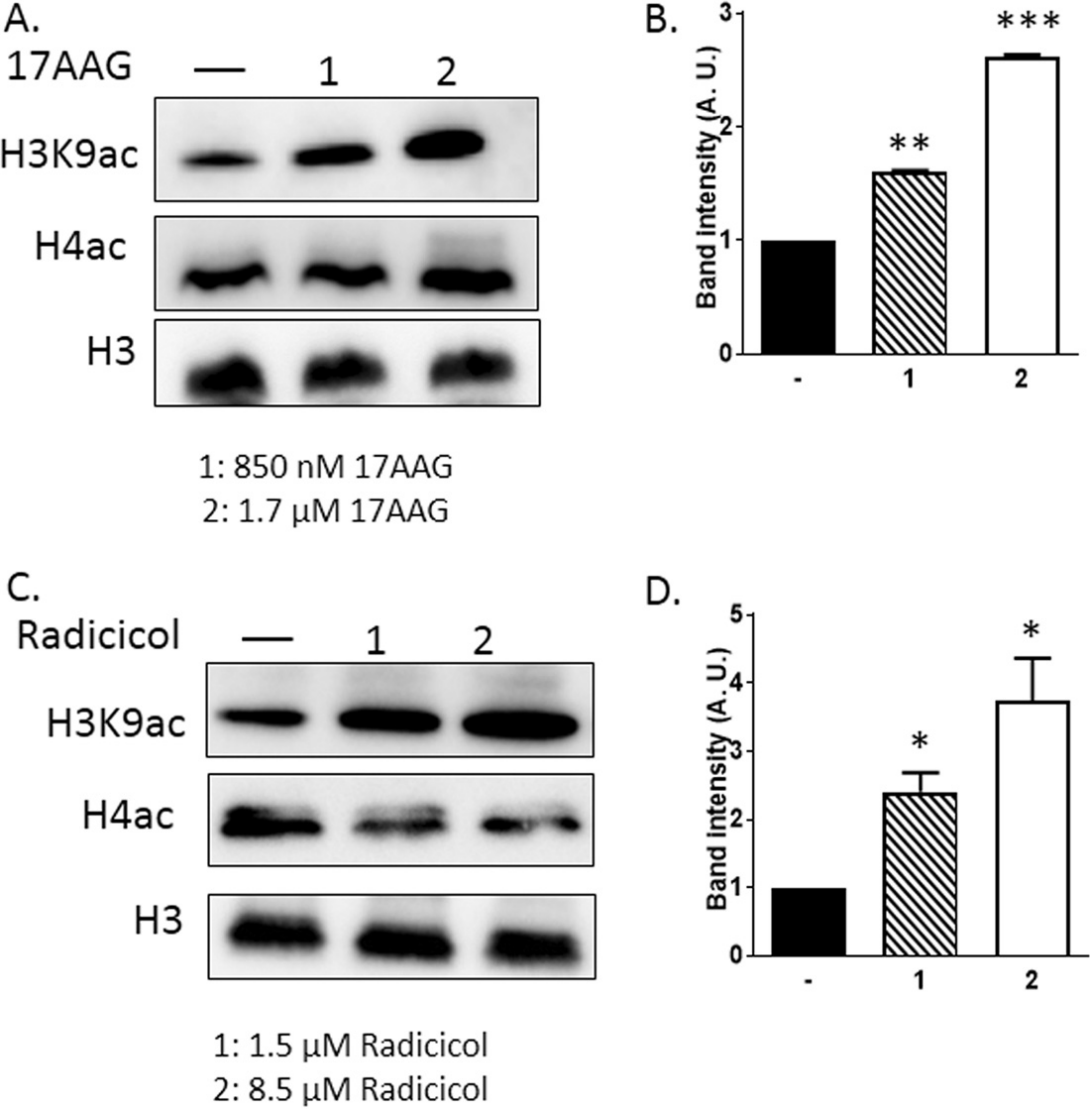
Chemical inhibition of PfHsp90 leads to hyperacetylation of histone H3 at K9 position. (A) Western blot analysis showing the level of acetylated H3K9 and H4 proteins performed from total protein isolated from untreated parasite or treated parasite with two different concentrations of 17-AAG as indicated. H3 was used as the loading control. (B) Graph exhibits the increase in H3K9ac levels upon Hsp90 inhibition by 17-AAG, plotted from the band intensities obtained from three individual experiments with error bar indicating the SEM. (C) Western blot analysis depicting the level of acetylated H3K9 and H4 performed from total protein isolated from untreated parasite or treated parasite with two different concentrations of Radicicol as indicated. H3 was used as the loading control. (D) Graph depicts the elevated level of H3K9ac upon Hsp90 inhibition by Radicicol. It was plotted from the values of band intensities obtained from three independent experiments with error bar indicating the SEM. The *P* value was calculated using the two-tailed *t* test (* means *P* value < 0.05; ** means *P* value < 0.01; *** means *P* value <0.001).

As PfHsp90-inhibitors affected PfSir2 functions at the rDNA locus and at the subtelomeric regions, we wanted to investigate whether such inhibition affects the histone deacetylase activity of PfSir2. PfSir2 is known to deacetylate the acetylated K9 residue of histone H3 (23, 25). We have used specific antibody to determine the acetylation status of total cellular H3K9Ac in the presence and absence of PfHsp90 inhibitors. We predicted that if PfHsp90 inhibitor affects the activity of PfSir2, there will be an increase in the steady-state level of H3K9Ac. Indeed, we observed a dose-dependent hyper-acetylation of H3K9Ac (Fig. 2A). As an earlier study used 850 nM and 1.7 μM 17-AAG to demonstrate the dose dependent inhibition of PfHsp90 activity, we have also used the same concentration of the drug (17). Upon treatment with 850 nM 17-AAG there was a 1.6-fold increment, while with 1.7 μM 17-AAG treatment there was more than 2.6-fold increment in the levels of H3K9Ac (Fig. 2B). No significant increase in the level of H4-Ac was observed under such conditions. The levels of total H3 protein were used to normalize those data. In order to ascertain that the observed effect is indeed due to the inhibition of PfHsp90, we have used another potent inhibitor of PfHsp90, namely, Radicicol. To this end, we treated the parasite culture with 1.5 μM and 8.5 μM Radicicol, as determined in an earlier study (15). A similar hyperacetylation of H3-K9 was observed upon treatment with Radicicol (Fig. 2C). With 1.5 μM and 8.5 μM Radicicol treatment, 2.4-fold and 3.7-fold increase in the level of H3K9Ac were observed, respectively (Fig. 2D). Also, the level of H4-Ac remained unaltered. Given that PfSir2A has been implicated in the deacetylation of H3K9Ac, these results suggest the possibility that such activity of PfSir2A could have been perturbed by the inactivation of PfHsp90.

### Chemical inhibition of PfHsp90 leads to depletion of PfSir2.

**FIG 3:**
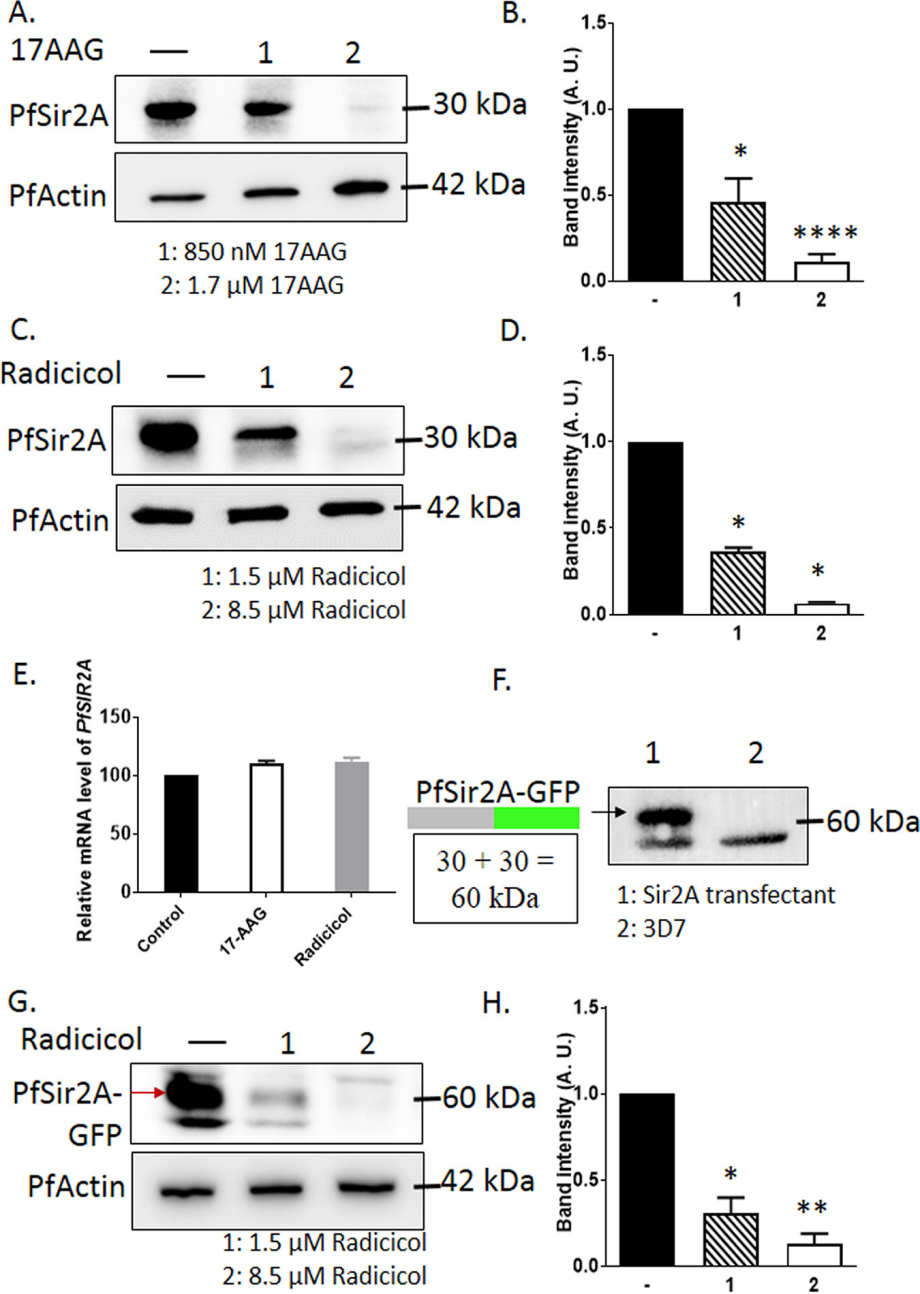
Chemical inhibition of PfHsp90 leads to the depletion of PfSir2A. (A) Western blot depicting the steady-state level of PfSir2A performed from total protein isolated from control and treated parasites with two different concentrations of 17-AAG as indicated. Actin was used as the loading control. (B) Graph was plotted from the values obtained from the band intensities of three independent experiments, depicting the depletion of PfSir2A in Hsp90 inhibitory condition. Error bar indicates the SEM (C) Western blot analysis showing loss of stability of PfSir2A performed from total protein isolated from control and treated parasites with two different concentration of Radicicol as indicated. Actin was used as the loading control. (D) Graph was plotted from the quantification band intensities obtained from three independent experiments with error bar indicating the SEM, showing the reduction in the abundance of PfSir2A upon PfHsp90 inhibition. (E) Graph indicating the real-time RT-PCR analysis showing mRNA levels of *PfSIR2A* in control, 17-AAG-, and Radicicol-treated conditions. *Seryl-tRNA synthetase* was used as the normalizing control and error bar indicates the SEM obtained from the three independent experiments. (F) Western blot analysis depicting the expression of PfSir2A-GFP in the transfected line but not in the untransfected 3D7 line. (G) Western blot showing a decline in the stability of PfSir2A-GFP in the Radicicol-treated parasites, performed from total protein isolated from PfSir2A-GFP overexpressing parasite lines. Actin was used as the loading control. (H) Graph has been plotted by quantification of band intensities obtained from three independent experiment, error bar indicates the SEM. The *P* value was calculated using the two-tailed *t* test (* means *P* value < 0.05; ** means *P* value < 0.01; **** means *P* value < 0.0001).

We sought to investigate whether the loss of PfSir2A activity under PfHsp90 inhibitory conditions is occurring due to the instability of the PfSir2A protein. If PfHsp90 is involved in the maturation of PfSir2A, inhibition of PfHsp90 might lead to the depletion or aggregation of PfSir2A protein. P. falciparum *in vitro* cultures at the ring stage were treated with various concentration of 17-AAG or Radicicol, and the steady-state level of PfSir2A protein was determined with a specific antibody. In a previous study investigating the dose dependent inhibition of PfHsp90 function in terms of providing the client stability, it was found that the treatment with 850 nM 17-AAG could result in a significant loss of PfRad51 protein, a client of PfHsp90, while a more dramatic effect was observed with 1.7 μM 17-AAG (16). The same study has also found that the treatment of 1.5 μM Radicicol could significantly lower-down the function of the PfHsp90 client PfRad51. Thus, we have used these doses of 17-AAG and Radicicol in our study. Dose dependent depletions of PfSir2A protein was observed with both17-AAG or Radicicol treatments (Fig. 3). Treatment with 850 nM 17-AAG resulted in 55% depletion, while with 1.7 μM 17-AAG almost a 90% depletion was observed (Fig. 3A and B). Similarly, treatment with 1.5 μM or 8.5 μM Radicicol resulted in a 67% and 99% depletion of PfSir2A protein, respectively (Fig. 3C and D). PfActin has acted as the loading control in both the cases.

In order to rule out the possibility that the reduced level of PfSir2A protein is not due to the transcriptional downregulation of *PfSIR2A* gene, we performed quantitative reverse transcriptase PCR (qRT-PCR) analysis with RNA isolated from inhibitor-treated and untreated parasite cultures. No significant change of *PfSIR2* transcript was observed upon treatments with 1.7 μM 17-AAG or 8.5 μM Radicicol (Fig. 3E).

In order to ascertain that the effect of PfHsp90 inhibition on PfSir2A is primarily at the protein level, we have generated a parasite line expressing a C-terminally GFP-tagged PfSir2A from an episomal plasmid. This was used to investigate the steady-state level of the fusion protein under PfHsp90 inhibitory condition (Fig. 3F). Using anti-GFP antibody, we observed that indeed there is a dose-dependent depletion of the PfSir2A-GFP fusion protein (Fig. 3G and H). This result suggests the depletion of PfSir2A protein due to the inactivation of PfHsp90 is not dependent on the chromosomal context. Taken together, these results imply PfHsp90 provides stability to the PfSir2A protein.

### PfHsp90 physically interacts with PfSir2.

**FIG 4:**
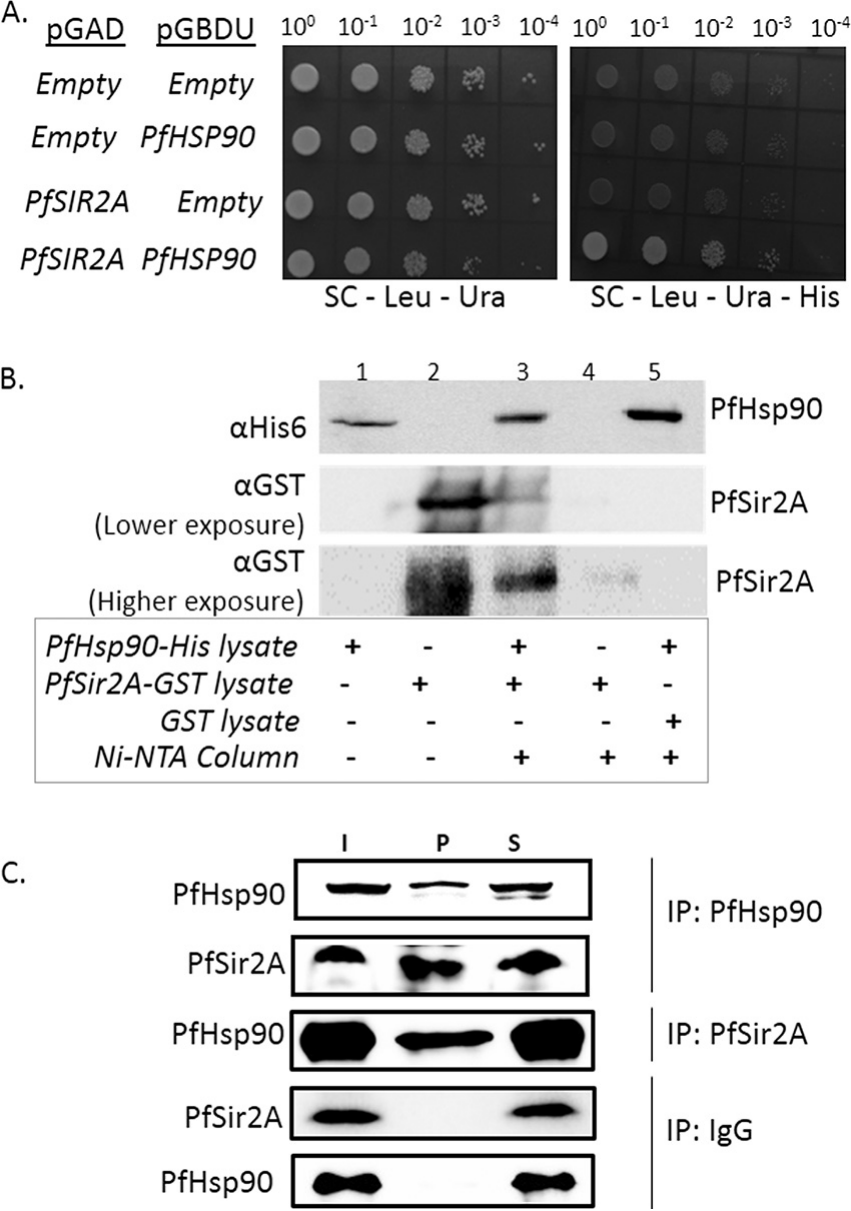
PfHsp90 physically interacts with PfSir2A. (A) Yeast-two-hybrid assay was performed to determine the interaction between PfSir2A and PfHsp90. *PfSIR2A* was cloned in the pGADC1 vector in order to fuse it to the *GAL4* activation domain (GAL4-AD) while *PfHSP90* was cloned in the pGBDUC1 vector and fused to *GAL4* DNA binding domain (GAL4-BD). PJ694a yeast strain harboring *HIS3* as reporter genes was used to perform the interaction studies. In order for spotting, the cells were grown until 0.5 OD_600_, and were then 10-fold serially diluted and spotted onto a plate lacking leucine and uracil to confirm the presence of the bait and the prey plasmids. The interaction was scored by spotting the cells onto triple dropout plates lacking leucine, uracil, and histidine. The assay was repeated three times and a representative image is shown (B). Western blotting showing interaction between PfHsp90 and PfSir2A by Ni-NTA pull-down (lane 3). Empty GST vector lysate does not show interaction when mixed with histidine-tagged PfHsp90 lysate (lane5). The lanes have been marked at the bottom. (C) Western blot analysis depicting the coimmnoprecipitation of PfHsp90 and PfSir2A using the parasite lysate. Anti-PfHsp90, anti-PfSir2A, and IgG antibodies were used to perform the IP. The blots were probed with anti-PfHsp90 or anti-PfSir2A antibodies, as indicated. The lanes are I, input; P, IP pellet fraction; and S, supernatant. The pellet fraction was loaded 12-times more (12×) than the input or supernatant fraction (1×).

We investigated whether there is a direct physical interaction between PfSir2A and PfHsp90 (PF3D7_0708400). If PfSir2A is indeed a direct client of PfHsp90, and the chaperone provides stability and functional maturation to PfSir2A protein, there must be a physical interaction between the chaperone and its client. An earlier high-throughput genome-wide yeast-two-hybrid screen has failed to score any interaction between PfHsp90 and PfSir2A (28). We have employed three different techniques to investigate the physical interaction between these two proteins: yeast-two-hybrid analysis, copurification assay with recombinant proteins, and coimmunoprecipitation (co-IP) experiment. For the yeast-two-hybrid analysis, *PfHSP90* was cloned in the bait vector as an in-frame fusion with the *GAL4* DNA binding domain, and *PfSIR2A* was cloned in the prey vector as an in-frame fusion protein with the *GAL4* activation domain. The presence of the bait and the prey plasmids were confirmed by the growth on the Leucine and Uracil drop-out plates. The protein-protein interaction was scored as the observed growth on the Histidine drop-out plates. As the yeast strain PJ69-4a is a Histidine auxotroph, lacking the endogenous *HIS3* gene, an activation of the *HIS3* reporter gene by virtue of the interaction between the bait and the prey proteins would only ensure growth on the Histidine drop-out plates. The observed robust growth on the triple drop-out plate is indicative of a physical interaction between PfHsp90 and PfSir2A (Fig. 4A). Empty bait or prey plasmids acted as the negative controls.

The interaction between PfHsp90 and PfSir2A was further confirmed via copurification assay. Recombinant His_6_-tagged PfHsp90 and Glutathione S-Transferae (GST)-tagged PfSir2A were expressed in bacteria and were then copurified on Ni-NTA affinity column. The presence of these proteins in the total lysate or in the purified fraction were detected by anti-His_6_ antibody or anti-GST antibody. The presence of both the proteins in the eluted fraction of the Ni-NTA affinity column suggested a direct physical interaction between PfSir2A and PfHsp90 (Fig. 4B).

In order to validate such interactions within the parasite, we have performed co-IP analysis. The parasite lysates from the schizonts stage were immunoprecipitated with anti-PfHsp90 antibody and the Western blots were probed with either anti-PfHsp90 or anti-PfSir2A antibodies. PfSir2A was found to be coprecipitated, suggesting a physical interaction with PfHsp90 (Fig. 4C). In a reverse co-IP experiment, PfSir2A was immunoprecipitated and the coprecipitated PfHsp90 was detected. In a parallel experiment, immunoprecipitation was done with control IgG and the Western blots were probed with anti-PfHsp90 or anti-PfSir2A antibodies. PfHsp90 or PfSir2A proteins were not detected in the pellet fraction, suggesting the specificity of the IP experiments. Thus, a physical interaction between PfSir2A and PfHsp90 was established.

## DISCUSSION

**FIG 5:**
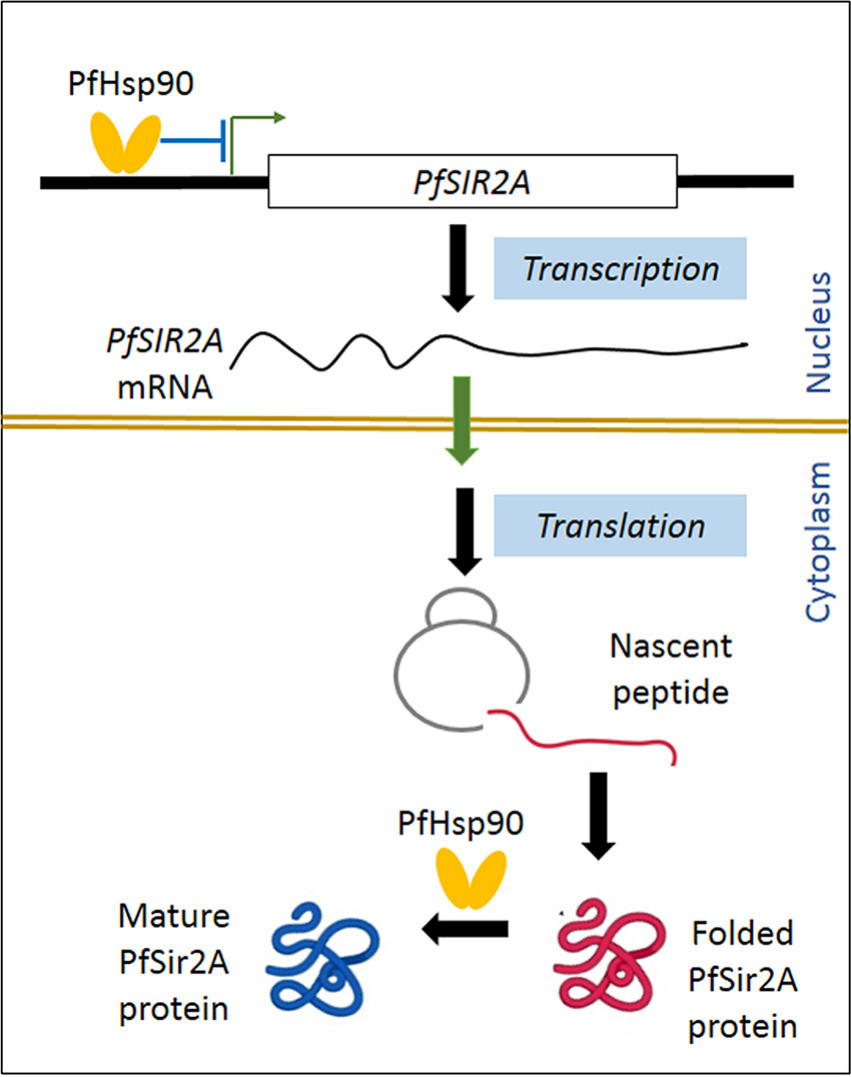
Model depicting the multifaceted roles of PfHsp90 in stabilizing and modulating the abundance and activity of PfSir2A. By virtue of its presence at the *PfSIR2A* promoter region PfHsp90 negatively regulates the transcription of *PfSIR2A* via its noncanonical nuclear function (27). Once PfSir2A is translated, PfHsp90 aids in the maturation of PfSir2A through its canonical chaperoning function.

The results presented here establish that the molecular chaperone PfHsp90 provides clientship to PfSir2A, thereby, modulating the abundance and activity of one of the major histone deacetylases in the human malarial parasite P. falciparum. First, a direct physical interaction between PfSir2A and PfHsp90 was observed. Second, if PfHsp90 is inhibited, a significant loss of PfSir2A activity was also observed, in terms of increased abundance of H3K9Ac, derepression of nucleolar rRNA transcription, and a loss of telomere-position effect at the *varA* loci. More importantly, inhibition of PfHsp90 resulted in a dose-dependent depletion of PfSir2A. Earlier, it was established that PfHsp90 negatively regulate the transcription of *PfSIR2A* in a developmental stage specific manner (27). Thus, it appears that PfHsp90 modulates the abundance and activity of PfSir2A in multiple ways (Fig. 5). It is a negative regulator of *PfSIR2A* transcription and a positive regulator PfSir2A protein stability. These findings underscore the tremendous importance of PfHsp90 in maintaining the protein homeostasis of PfSir2A.

Our finding emphasizes the evolutionary conservation of the clientship relation between Hsp90 and Sir2 in eukaryotes. Such clientship was first discovered in the budding yeast S. cerevisiae (29), although a direct physical interaction between ScSir2 and ScHsp90 could not be established. However, an interaction between a mutant ScHsp82^E33A^ and ScSir2 was noted (2). Later, the Sir2 orthologues from C. elegans, and humans, namely, SIRT1, were also found to be the clients of their respective Hsp90 counterparts (30). Interestingly, in another protozoan parasite, Leishmania major, a physical interaction between LmSIR2(RP1) and Hsp83 was established; however, the biological importance of such interaction remained elusive (31).

It was observed in our study that the effect of PfHsp90-inactivation mediated depletion of PfSir2 protein is not uniform across all the *var* genes tested. Such locus specific effect of Sir2 depletion was also observed in the budding yeast. It was found that the derepression at the mating-type locus was more prominent compared with the derepression at the subtelomeric locus (29). Currently, the reason behind such phenomenon is not clearly understood. It could be possible that some other compensatory mechanisms, including nuclear architecture and nuclear territories, may mask the loss of *var* silencing due to the depletion of PfSir2. This possibility seems plausible, as it is well-established that a direct correlation between PfSir2 level and *var* silencing does not hold true in several P. falciparum strains (32). Future studies might unravel the molecular basis of such observations.

Given the multifaceted roles of PfSir2 in *Plasmodium* biology, and the nondispensable functions of PfHsp90, coupled with our current findings that the cellular levels of PfSir2A is maintained by PfHsp90, it is reasonable to propose that the PfSir2A-PfHsp90 axes could be explored as a novel target to curb malaria.

## MATERIALS AND METHODS

### Plasmids.

The plasmid pGBDUC1:*PfHSP90* used for the yeast two hybrid analysis has been previously described (17). *PfSIR2A* was amplified using the forward primer OMKB18 (CGATGGATCCATGGGTAATTTAATGATTTCC) and reverse primer OMKB2 (TCCGGAGCTCTATGTGTGCGTGTGAGCTAC) with P. falciparum 3D7 genomic DNA as the template. It was then cloned in the pGADC1 vector. For the copurification experiment, *PfHSP90* was cloned and expressed from the pCold-I vector. To this end, *PfHSP90* gene was amplified from cDNA with the primer-sets OMKB469 (TCA GGA TCC ATG TCA ACG GAA ACA TTC GC) and OMKB 470 (TCA GTC GAC ATC CTT TAG TCA ACT TCT TCC) and then cloned in the pCold-I vector. Plasmid pGEX6p2:PfSir2A, used for the recombinant expression of *PfSIR2A*, has been previously described (27). For transfection, *PfSIR2A* was amplified with the primers OMKB622 (CCG GGT ACC ATG GGT AAT TTA ATG ATT TCC) and OMKB 623 (CCG CCT AGG CAT TAT TTT CTT ATT TTT TTC AC) using P. falciparum 3D7 genomic DNA as the template and then cloned in the pARL vector (33).

### Antibody used.

Anti-PfSir2A antibodies were generated in our laboratory (27). The anti-Actin antibody (Cat: A5441, Sigma, St. Louis, MO, USA) recognizes PfActin as previously described (34). The anti-Hsp90 antibodies (Cat: H1775, Sigma, St. Louis, MO, USA) recognize PfHsp90 protein as previously described (27). The anti-GFP antibodies (Cat: ab290, Abcam, Cambridge, UK), anti-H3 (Cat 9715s Cell Signaling), anti-H3K9ac (Cat: 07-353, Millipore), anti-H4ac (Cat: 06-866, Millipore), the HRP conjugated anti-rabbit, and anti-mouse secondary antibodies (Promega, Madison, WI, USA) were used in this study.

### Parasite culture and synchronization.

P. falciparum 3D7 cultures and PfSir2A transfectant line were maintained by the candle jar method at 37°C as described previously (35) at 5% haematocrit, with 1% albumax (Invitrogen, Carlsbad, CA, USA) and 0.005% hypoxanthine supplemented RPMI 1640 medium. Parasite cultures were synchronized at the ring stage by treatment with 5% sorbitol (Sigma, St. Louis, MO, USA).

### Transfection in P. falciparum.

Hundred micrograms of pARL:PfSir2A plasmid was transfected into synchronous ring stage parasites with 6% to 8% parasitemia following the protocol described previously (36). Briefly, a day before the transfection, the plasmid was resuspended in cytomix (10 mM K_2_HPO_4_ [pH 7.6], 120 mM KCl, 0.15 mM CaCl_2_, 25 mM HEPES [pH 7.6], 2 mM EGTA [pH 7.6], and 5 mM MgCl_2_) and stored at 4°C overnight. Electroporation for transfection was performed using a Bio-Rad gene pulser. After the transfection, parasites were gown in complete media and were supplemented with Pyrimethamine drug media once the parasitemia reaches around 4%.

### 17-AAG and radicicol treatment.

The 17-AAG (Sigma, St. Louis, MO, USA) and Radicicol (Sigma, St. Louis, MO, USA) treatments were given to the synchronous parasite cultures at the ring stage. Parasites were harvested at the late trophozoite/early schizont stage for RNA isolation and protein preparation.

### Western blot analysis.

The protein samples were separated on 12% SDS Polyacrylamide gel and subjected to Western blotting as previously described (37). Anti-PfSir2A antibodies raised in rabbit were used in 1:10,000 dilution, while all the other primary antibodies anti-Actin (raised in mouse), anti-H3 (raised in rabbit), anti-H3K9ac (raised in rabbit), anti-H4ac (raised in rabbit), anti-Hsp90 (raised in mouse), and anti-GFP (raised in rabbit) were used in 1:5,000 dilution. The HRP conjugated secondary antibodies anti-mouse and anti-rabbit were used in 1:10,000 dilution. The protein signal was detected using Enhanced chemiluminescence (ECL) kit (Pierce). Image J software was used to determine the band intensities and the graphs were plotted using the Graph pad prism 6 software.

### Co-IP.

Co-IP was performed as previously described (17) using the Pierce Crosslink Immunoprecipitation Kit (Thermo Scientific). For determining the interaction between PfHsp90 and PfSir2A, pull-down was performed by cross-linking anti-PfSir2A, or anti-Hsp90 to Pierce Protein A/G Plus Agarose beads. For the negative control, beads were cross-linked to anti-IgG for performing the pull-down.

### Yeast-two-hybrid.

Yeast-two-hybrid (Y2H) analysis was performed as described earlier (37). The bait and the prey plasmids with and without the gene of interest were transformed in the yeast strain PJ694a. To score for the interaction, the expression of the reporter gene *HIS3* was monitored. The transformed PJ694a cells were serially diluted 10-fold and plated on triple dropout SC-leu-ura-his plates. The cells were also plated in the double drop out plate SC-leu-ura as a control.

### Copurification.

For performing the copurification analysis, the BL21(DE3) cells harboring pCold I-PfHsp90 was induced using 1 mM IPTG at 15°C for 24 h, while the Codon Plus cells harboring pGEX6p2-PfSir2A or pGEX6p2 empty vector were induced as described previously (38). Lysis and Ni-NTA purification were performed similarly as previously described (39). Briefly, the lysates from the two bacterial cultures expressing either His_6_-PfHsp90 or GST-PfSir2 were combined together before applying to the Ni-NTA column. The proteins were eluted by 250 mM Imidazole treatment. The cells expressing only GST tag served as a negative control. The elute samples obtained were subjected to Western blot analysis.

### RNA isolation and real-time PCR.

The total RNA was isolated as previously described (27). The concentration of the isolated RNA was measured by spectroscopic analysis. Five microgram of RNA was subjected to DNase treatment for removal of any DNA contamination. Prior to cDNA synthesis, PCR analysis was performed (– RT) to ensure the complete removal of genomic DNA. For synthesis of cDNA, 1 μg of RNA was reverse transcribed using oligo dT primer (Sigma, St. Louis, MO, USA) and Omniscript reverse transcriptase (Qiagen, Hilden, Germany). The synthesized cDNA was subjected to real-time analysis using the SYBR premix Ex taq (TaKaRa Bio, Kusatsu, Shiga, Japan). Applied Biosystems 7500 fast real-time PCR machine was used for the q-PCR analysis. The primers used and real-time analysis for *var* genes were described previously (40). Primers used for reverse tranccriptase PCR (RT-PCR) of ribosomal locus were previously listed (24). *Seryl-tRNA synthetase* was used as the normalizing gene.
